# Older Adults’ Perspectives on Participating in a Synchronous Online Exercise Program: Qualitative Study

**DOI:** 10.2196/66473

**Published:** 2025-04-03

**Authors:** Giulia Coletta, Kenneth S Noguchi, Kayla Beaudoin, Angelica McQuarrie, Ada Tang, Rebecca Ganann, Stuart M Phillips, Meridith Griffin

**Affiliations:** 1 Department of Kinesiology, McMaster University, Hamilton, ON, Canada; 2 School of Rehabilitation Science, McMaster University, Hamilton, ON, Canada; 3 School of Nursing, McMaster University, Hamilton, ON, Canada; 4 Department of Health, Aging and Society, McMaster University, Kenneth Taylor Hall, 241, 1280 Main Street West, Hamilton, ON, L8S 4M4, Canada, 1 905-525-9140 ext 27417; 5 Gilbrea Centre for Studies in Aging, McMaster University, Hamilton, ON, Canada

**Keywords:** exercise, older adults, qualitative study, qualitative, experience, attitude, opinion, perception, perspective, interview, internet, kinesiology, physiotherapy, synchronous, online, home-based, gerontology, geriatric, older, aging, physical activity

## Abstract

**Background:**

Older adults face several barriers to exercise participation, including transportation, lack of access, and poor weather conditions. Such barriers may influence whether older adults meet the Canadian 24-Hour Movement Guidelines. Recently, older adults have adopted technology for health care and are increasingly using digital health technologies to improve their access to care. Therefore, technology may be a valuable tool to reduce barriers to exercise and increase exercise participation rates within this population.

**Objective:**

This study aimed to explore older adults’ perceptions and experiences of exercise, in general, and specifically related to our synchronous online exercise program for community-dwelling older adults.

**Methods:**

A total of 3 registered kinesiologists and 1 physiotherapist with experience working with older adults delivered an 8-week, thrice-weekly synchronous online group-based exercise program for older adults in 3 cohorts. The program focused on strength, balance, and aerobic activity. Following the program, a qualitative study with interpretive descriptive design was conducted to explore participants’ perceptions and experiences. Participants were invited to take part in a 30-minute, one-on-one semistructured interview via Zoom with a research team member. Interview data were thematically analyzed to identify common themes.

**Results:**

A total of 22 older adults (16 women, 6 men; mean age 70, SD 4 years) participated in interviews. Three themes were identified as follows: (1) health, exercise, and aging beliefs; (2) the pandemic interruption and impacts; and (3) synchronous online exercise programs attenuate barriers to exercise. Participants discussed their exercise beliefs and behaviors and their desire to safely and correctly participate in exercise. Older adults found that their physical activity was curtailed, routines disrupted, and access to in-person exercise programs revoked due to the pandemic. However, many suggested that our synchronous online exercise program was motivational and attenuated commonly reported environmental barriers to participation, such as transportation concerns (eg, time spent traveling, driving, and parking), accessibility and convenience by participating at a location of their choice, and removing travel-related concerns during poor weather conditions.

**Conclusions:**

Given these reported experiences, we posit that synchronous online exercise programs may help motivate and maintain adherence to exercise programs for older adults. These findings may be leveraged to improve health outcomes in community-dwelling older adults.

## Introduction

Physical activity (PA) guidelines are not met by ≈85% of older adults in Canada [1,2]. The Canadian Society for Exercise Physiology recommends that older adults participate in 150 minutes of moderate-to-vigorous PA and 2 bouts of strengthening exercise each week [3], similar to the World Health Organization guidelines [4]. If PA guidelines are not met, older adults are at greater risk of adverse health outcomes, including falls, cardiovascular disease, mobility limitations, hospitalization, and mortality [4].

Older adults commonly report barriers to participating in PA and exercise. A systematic review by Spiteri et al [5] determined that environmental factors, resources, lack of assistance in managing change, and social influences are among the common barriers to PA participation for older adults aged 65‐70 years. Baeret et al [6] reported similar barriers in their systematic review of 44 journal papers describing motivators and barriers to PA in older adults. Common environmental barriers for older adults include transportation, geographic accessibility, affordability, and weather [5-7]. Transportation barriers can be attributed to a lack of transportation to facilities, challenges navigating parking, and poor reliability of affordable transportation [5,6]. Lack of access to exercise facilities, the cost of exercise programming, and distance to programs contribute to environmental barriers [5,6]. Lastly, poor weather conditions reduce the likelihood of adults participating in recreational activities or attending recreation centers [7]. Among Canadians, 64% are less active in the winter compared with the summer [8]. Developing exercise programs to mitigate common environmental barriers may aid in improving older adults’ abilities to participate in exercise and meet PA guidelines.

Recently, older adults have adopted technology for health care to improve access to care [9,10]. Technology may, therefore, be a valuable mode of delivery to reduce barriers to exercise and increase participation rates within this population [11]. Current evidence suggests that online exercise programs improve physical function and cognition in older adults, although these programs are exclusively asynchronous, web-based, or interactive exercise-based video games [12]. Older adults have reported not enjoying asynchronous exercise programs as they lack social connectivity, accountability, and adherence, making it difficult to stay motivated [12,13]. Recently, there has been a greater uptake of asynchronous and synchronous programming due to the COVID-19 pandemic [14-16]. A scoping review by Dagenais et al [16] described the nature and extent of the existing literature on online exercise programming among older adults and found 17 studies using asynchronous (9/17, 53%), synchronous (5/17, 29%), or both asynchronous and synchronous programming (3/17, 18%). For older adults, group-based synchronous programs have demonstrated improvements in muscle strength, balance, physical function, aerobic capacity, and quality of life [14]. Therefore, gaining insights into older adults’ perceptions and experiences with synchronous programming is important.

We developed and delivered a synchronous online exercise program to help older adults engage in exercise while in-person programming was temporarily suspended [17]. The synchronous program was designed and delivered by registered kinesiologists and physiotherapists. The main aim of the parent trial was to investigate the preliminary effectiveness and feasibility of the synchronous online exercise program through a mixed method pilot randomized controlled trial (RCT) [17]. The aim of this paper was to explore older adult participants’ perceptions and experiences of exercise, in general, and of our synchronous online community exercise program.

## Methods

### Design

This qualitative study was part of an 8-week community-based, pilot RCT [17]. An interpretive descriptive design explored community-dwelling older adults’ experiences and perceptions of exercise, in general, and of the synchronous online exercise program [18]. Interpretive description offers a flexible approach to analyzing qualitative data within medical education research, as it can explore individuals’ experiences while producing practical clinical outcomes [19]. The pilot RCT compared community-dwelling older adults aged 65 to 80 years participating in a synchronous online exercise program (ACTIVE) delivered on Zoom with a waitlist control group (CON). A total of 3 registered kinesiologists and 1 physiotherapist with experience working with older adults conducted the intervention online in 3 cohorts at the Physical Activity Centre of Excellence at McMaster University in Ontario, Canada. The intervention and all data collection, including the qualitative interviews, were conducted remotely via Zoom. Zoom meets McMaster University’s privacy legislation. The study duration was from January 2021 to May 2022. The study was reported in accordance with the consolidated criteria for reporting qualitative research [20]. For the purpose of this paper, the qualitative findings alone will be reported.

### Ethical Considerations

This study was approved by the Hamilton Integrated Research Ethics Board (#11429) and registered at ClinicalTrials.gov (NCT04627493). Participants were informed of the study content and procedures both verbally and in writing. Informed written and oral consent was obtained from all participants. All participants had the right to withdraw from the study at any time without any adverse consequences. All data were anonymized. Participants did not receive compensation for their participation.

### Participants

ACTIVE and CON group participants enrolled in our 8-week synchronous online program were invited via email to participate in an individual qualitative interview after completing the exercise program. The eligibility criteria for the full trial have been previously reported [17]. Briefly, they were healthy older community-dwelling women and men between the ages of 65 and 80 years, had access to the internet via a personal smartphone, tablet, or computer, and participated in ≤150 minutes of moderate to vigorous PA per week. To be eligible for the qualitative portion of the study, participants must have remained enrolled in the study and needed to participate in the online exercise program. Interested participants were contacted within 2 weeks of the end of the synchronous online exercise program to schedule an interview time. With a purposeful subsample of interested participants from the pilot RCT, we aimed to conduct between 20 and 24 interviews, as data saturation typically occurs within this range [21].

### Intervention

The 8-week intervention consisted of thrice-weekly 60-minute synchronous online exercise classes (totaling 24) delivered online via Zoom by health professionals (ie, registered kinesiologists and physiotherapists). Briefly, the group-based classes began with a 5-minute warm-up, 50 minutes of progressive strength (25 min), aerobic (20 min), and balance training (5 min), and a 5-minute cool-down [17]. The physiotherapist (n=1) and registered kinesiologists (n=3) were trained to work with older adults and adjusted exercise intensity weekly to meet participants’ abilities. The instructors and research team members supervised and moderated each class to ensure participant safety and assist with technological difficulties. All interactions between participants and the research team were conducted remotely via Zoom; no pre-established relationships existed prior to the study.

### Qualitative Data Collection

Semistructured, open-ended one-on-one interviews were conducted via Zoom by a study investigator (GC, female, PhD Candidate) with experience in qualitative research [22]. A qualitative interview guide (Multimedia Appendix 1) was developed with a qualitative methodology expert (MG) to generate discussion around participants’ experiences and perceptions of exercise, in general, and with our synchronous online exercise program [22]. Questions were not provided to participants before the interview. However, participants were aware of the interviewer’s personal goals (ie, PhD research) for conducting the research. Transcripts were transcribed verbatim from Zoom audio recordings, checked for accuracy, and not returned to participants.

### Data Analysis

The interviews were thematically analyzed using the computer software Dedoose (version 9.0.17; SocioCultural Research Consultants, LLC) [23]. Two authors, GC and KSN, independently coded the first 2 transcripts and discussed initial patterns and themes to inform the preliminary coding book [24], which was shared and refined with input from the qualitative expert. The coding book was used to review subsequent transcripts and iteratively revised to reflect new concepts. Themes and subthemes were inductively developed and refined with the research team. At least 2 interviewers (GC, KSN, and MG) discussed any disagreements in transcription or content to reach a consensus [23,25].

## Results

### Participants’ Characteristics

Of the 32 who participated in the pilot RCT, which determined the preliminary effectiveness of the synchronous online exercise program [17], 22 older women and men participated in semistructured interviews. Ten participants declined or did not respond to the invitation to participate in this study. Table 1 presents the descriptive characteristics of the sample. There were no major differences between groups for any variable. The majority of participants were women (25/32, 78%) and had completed postsecondary education (29/32, 91%). All interviews were approximately 30 minutes in duration by design.

**Table 1.** Descriptive characteristics for the ACTIVE, control group (CON), and no-interview participants (N=32).

	ACTIVE^a^ (n=13)	CON^b^ (n=9)	No interview (n=10)
Women, n (%)	9 (69)	7 (78)	9 (90)
Age (years), mean (SD)	70 (4)	70 (4)	74 (4)
Race, n (%)			
Caucasian	12 (92)	9 (100)	9 (90)
Indigenous	1 (8)	0 (0)	0 (0)
Asian	0 (0)	0 (0)	1 (10)
Highest level of education earned, n (%)			
Some school or high school diploma	2 (15)	1 (11)	1 (10)
Some college, vocational, or training school	1 (8)	1 (11)	0 (0)
College graduate or bachelor’s degree	5 (38)	5 (56)	7 (70)
Postgraduate training or degree	5 (38)	2 (22)	2 (20)
Partner status, n (%)			
Never married	0 (0)	2 (22)	0 (0)
Divorced or separated	4 (31)	1 (11)	1 (10)
Widowed	3 (23)	1 (11)	3 (30)
Presently married	6 (46)	5 (56)	6 (60)
Living arrangement, n (%)			
Wife, husband, or partner	6 (46)	5 (56)	6 (60)
Children	1 (8)	0 (0)	0 (0)
Friends	0 (0)	1 (11)	0 (0)
Living alone	5 (38)	3 (33)	4 (40)
Other	1 (8)	0 (0)	0 (0)
Physically active prior to the pandemic, n (%)	7 (54)	5 (56)	6 (60)

a ACTIVE: exercise group.

b CON: waitlist control group.

### Qualitative Findings

#### Overview

Our thematic analysis identified 3 main themes with respect to participants’ perceptions of exercise in general and with the synchronous online exercise program: health, exercise, and aging beliefs; the pandemic interruption and impacts; and synchronous online exercise programs attenuate barriers to exercise (Textbox 1). Participants described various subthemes within these 3 main themes, including their exercise beliefs and behaviors and their desire to safely and correctly participate in exercise as an older adult. They also reflected upon the impact of the pandemic on their PA, routine, and lack of access to exercise programs. Participants discussed how the online synchronous format contributed to their motivation to exercise, reduced concerns about transportation, enabled participation from their desired training location, and allowed them to avoid poor weather conditions. Our findings provide greater insight into older adults’ perceptions and experiences of exercise, in general, through the themes of health, exercise, and aging beliefs and the impact of the pandemic on older adults’ PA. We also provide insight into the delivery of synchronous online community exercise programs and how common barriers to exercise may be attenuated.

Textbox 1. **Qualitative themes and subthemes.**

**Health, exercise, and aging beliefs**

- Exercise beliefs and behaviors.
- Desire to safely and correctly participate in exercise as an older adult.

**The pandemic interruption and impacts**

- Physical activity curtailed.
- Routines disrupted.
- Access to exercise programs revoked.

**Synchronous online exercise programs attenuate barriers to exercise**

- Synchronous programming is motivating.
- Removed concerns about transportation.
- Ease of participation from desired training location.
- Removed poor weather condition concerns.

#### Health, Exercise, and Aging Beliefs

Participants believed in the benefits of exercise and sought ways to become more physically active. Many had participated in PA throughout their lives, and they valued the importance of the health benefits of exercise. Three participants described their beliefs about the benefits of exercise and its importance to health and longevity.

*Yeah, it’s like exercise should be long-term or lifetime commitment… it can really benefit you through your own lifetime.*

[CON1]

*I have the attitude exercise is important for everybody, but it’s especially important as we get older.*

[CON2]

*I need physical exercise. My body feels better.*

[CON3]

Two participants discussed the reasons for seeking out opportunities to increase their PA.

*If I joined the program at least it would give me a reason to get up and get started in exercise.*

[ACTIVE4]

*Since I’ve retired, I’m trying to find some kind of routine that would keep me active, and [help with] losing weight and trying to get healthy for longevity.*

[ACTIVE5]

Participants expressed concern about their declining fitness levels. Three participants described their desire to continue participating in meaningful activities and believed that becoming more active would help them maintain or improve their health.

*I was getting too unfit basically… If I think in terms of basic fitness, it would be functional fitness. Can I bend down to get something on the bottom shelf? Can I reach the top shelf? Can I get in and out of a bathtub?… Activities of daily living, but all of them, like the reaching bending stretching [exercise would help with this].*

[CON6]

*Because I want to be active in my older age. I don’t want to be, you know, stuck in a chair and very limited in what I can do. I still want to travel… You know I want to be mobile.*

[ACTIVE7]

*What does motivate me is as I’m aging, I want to get healthier… I want to maybe see my grandkids get married, you know, things like that, so as I age, I get more motivation.*

[ACTIVE8]

One participant described their motivation to become more physically active for health and vanity.

*It’s for health, and to be honest with you, it partly is vanity, too.*

[CON2]

The participants in our study had prior beliefs regarding exercise and believed in the long-term benefits of exercise. Their motivation stemmed from their desire to improve their health and continue to participate in activities that are meaningful to them. While participants expressed a desire to be active, many disclosed that they lacked knowledge of how to exercise. One participant described that as an older woman, strength training is not an easily accessible exercise modality. Aerobic is traditionally the commonly prescribed type of exercise, but older adults, including women, are interested in learning about strength training.

*I think as an older person and a woman, I’m less likely to do [strength training] on my own. I can go out and walk and do aerobic stuff, but it’s the resistance work that I would be interested in.*

[ACTIVE5]

Three participants described concerns over incorrect form, potentially leading to injury. Older adults discuss a lack of confidence, fear of injury, and desire for more supervision when exercising.

*I think as we get older, certainly doing it the proper way even becomes more important than when you were young.*

[ACTIVE9]

*It’s not like I didn’t want to try and join some of those [traditional] gyms sometimes, but I didn’t feel as confident about it because of some of the medical part [injuries].*

[CON3]

*But there’s not a lot of supervision happening there [traditional gyms]… the opportunity to do things incorrectly kind of opens you for doing something wrong and hurting yourself.*

[ACTIVE7]

Participants in this study desired to become more physically active and believed in the benefits of exercise. Their motivations for exercising varied from wanting to continue with their activities of daily living to longevity and vanity. However, our participants suggested that traditional gym settings do not offer adequate supervision for older adults as they learn to participate in exercise safely.

#### The Pandemic Interruption and Impacts

The COVID-19 pandemic interrupted participants’ daily routines and limited access to PA during this study. The first cohort of participants began the study during the second wave (Beta variant) of the COVID-19 pandemic and completed the study during the third wave (Gamma variant). The second cohort of participants began the study during the third wave of the COVID-19 pandemic and completed the study as the fourth wave (Delta variant) began. The third cohort of the study started and completed the study during the fifth wave (Omicron variant) of the COVID-19 pandemic. Participants voiced several concerns about their own inactivity levels and how these were affected by COVID-19. For example, 3 participants spoke of the secondary consequences of the pandemic resulting in unhealthy habits.

*I had joined the gym, and then COVID happened. So, then I’ve put on another 30 pounds, so now I’m 70 pounds over my optimum weight…. I don’t want to risk it right now because of the COVID stuff. Yeah, and I’m not doing massages and not doing physiotherapy, so you know that’s kind of hindered things too.*

[ACTIVE10]

*I got out first thing in the morning; it was a part of a routine during the pandemic. I lost all of that; consequently, I put on weight… when the pandemic hit, I literally turned into a couch potato.*

[CON2]

*I was really surprised at how de-conditioned I became over the course of the pandemic; I mean, it wasn’t great before, but it became a lot worse during the pandemic.*

[ACTIVE11]

The pandemic’s secondary consequences left participants more sedentary during this period and disrupted their routines. For example, 2 participants described the challenge to keep track of weekdays.

*COVID really slowed us down, and I didn't know what day of the week it was.*

[CON1]

*Well, yeah, until the program that you gave us, I was getting lost on what day of the week it was. At least I knew when it was Monday, Wednesday, Friday.*

[ACTIVE10]

In addition to disruptions to participants’ daily routines, the pandemic revoked access to exercise programs. The public health-mandated lockdowns resulted in canceled in-person exercise programs, further limiting access to PA and exercise. Prior to the pandemic, participants had been engaging in exercise programs and were members of local gyms. COVID-19 interrupted their participation in programming, and many found it difficult to motivate themselves to exercise.

*… once COVID hit, other classes [exercise programs] closed, it was very difficult… it’s almost impossible to continue [exercising]. And I’m not very good at self-motivating.*

[CON1]

*Okay, I go to the gym quite often, like maybe four times or five times a week and, of course, the gyms are closed [COVID], and I found it hard to get motivated to do stuff on my own.*

[ACTIVE4]

*I just started at the Mac gym in the Senior’s Program in February, so I was just sort of getting into the three-times-a-week schedule when, of course, we stopped in March because of the pandemic.*

[ACTIVE11]

The pandemic interrupted many participants’ routines, including canceled exercise programs and memberships and curtailing PA.

#### Synchronous Online Exercise Programs Attenuate Barriers to Exercise

Participants reported that our synchronous online exercise program removed several common environmental barriers to exercise and motivated them to become more physically active. For example, one participant discussed enjoying exercising from home while still connecting to people in real time.

*The fact that we got to do it at home… that was cool. I think, maybe something like that would probably motivate me again that there’s interaction, it’s real people, and it’s done in real-time… I actually liked having it [online].*

[ACTIVE8]

Another participant spoke about how synchronous programming, in general, allows them to continue to exercise when they are not comfortable attending in-person programming.

*Yeah, if you have sniffles, you might still want to do the exercises, but you don't want to spread the disease…*

[ACTIVE10]

A third participant described how our synchronous online exercise program was advantageous compared with in-person programming.

*So, it’s just me moving around in the room, not, you know, bumping into other people like in a small gym… We’re always overheated. Here I have a fan blowing on me, and I don’t have somebody complaining that wind is blowing on them because they don’t like wind.*

[ACTIVE10]

Our synchronous online exercise program motivated participants to exercise as it allows individuals to remain connected with others while participating safely and easily from their own homes. Participants described enjoying the ease of participating in our program from home since it removed their concerns about transportation and reduced time spent traveling to and from the exercise program, which allowed more time to engage in other activities. Participants discussed their concerns about driving, traffic, and parking when attending in-person exercise programs at the university or recreational facilities. For example, one participant referred to the difficulty of driving and finding their way around campus.

*… I didn’t like driving to McMaster, and then you have to figure out where you’re going… that was just a part that I don’t care about… So, I like local these days, and the fact that I could do that on Zoom is great. I think it’s the best thing ever.*

[CON12]

Another participant spoke about not having to drive (or otherwise find their way) to the session and how it streamlined their exercise experience.

*No, that was actually really, really good. So, because it’s COVID, you don’t have to worry about being in contact. But yes, I don’t like the concept of having to drive somewhere to exercise. It just defeats the whole purpose… I liked that.*

[ACTIVE5]

A third participant discussed the inefficiency of driving to the gym.

*I always used to say if people just walk to the gym or bike to the gym they wouldn’t even have to go in the gym. So yes, you’ve cut out that timepiece of having to go somewhere to do something.*

[ACTIVE7]

Our online exercise program eliminated transportation concerns and was described as motivational as it allowed individuals to participate from their desired training location, including their homes and while on holiday. For example, one participant described the ease of connecting to our program while away from home.

*I was out of town, okay? I ended up at my nephew’s place, and he works the night shift. So, I didn’t want to disturb him inside, so I went outside, and they had a nice strong Wi-Fi connection, and it worked out really good.*

[CON1]

Another participant enjoyed the flexibility of our program, allowing them to maintain their routine while away from home.

*We’ll sign up for that. We can do it at the lake as well… With this type of activity, you can do it in your living room, you can do it out on the deck, and you can do it wherever you want. I think it’s great that there’s more flexibility, that you can take the program to where you are, rather than you go[ing] there.*

[ACTIVE9]

Two participants discussed how offering the exercise program through a synchronous videoconference platform enabled participation due to the ease of access.

*It [Zoom] made me kind of want to go on… how can you argue about getting up five minutes before it starts? And, yeah, it’s really, it’s fantastic.*

[ACTIVE13]

*Having it be virtual means that you don't have to worry about traffic and parking and driving.*

[CON6]

The synchronous program allowed flexibility in how and where participants engaged with our program, easing participants’ concerns about traveling in poor weather conditions. Older adults described their fears and worries about navigating poor weather conditions (eg, extreme heat, rain, snow, and ice) on foot or by car to attend exercise programs or to be physically active. One participant described enjoying not needing to leave the house during the winter months to exercise.

*Yeah, absolutely. In the winter, too, you didn't have to, you know, shiver and so on.*

[CON14]

Two participants spoke of the inconvenience of navigating the weather conditions when exercising.

*To go warm up my car, put my boots on, then go to the gym, take my boots off... It just doesn’t make sense to me.*

[ACTIVE8]

*It was helpful that I didn't have to go running and try to park somewhere and find something [parking] and go through a snowbank.*

[CON3]

Another participant discussed barriers when leaving the house when weather conditions are poor and how exercising from home helped to ensure they could still exercise.

*… if it’s like a torrential rain or snowstorm or ice, I don’t go out on ice. Yeah, you know, then you would do it from home.*

[6ACTIVE10]

Participants described poor weather conditions as negatively impacting their PA levels. Overall, they enjoyed the ease of the videoconference platform and participating in a synchronous online exercise program.

## Discussion

### Principal Findings

This study provides novel insights into synchronous online exercise programs for older adults. Specifically, participants identified themes including health, exercise, and aging beliefs; the pandemic interruption and impacts; and synchronous online exercise programs attenuating barriers to exercise*.* Our findings suggested that participants in this study believed in the benefits of exercise and wanted to learn how to exercise safely. The pandemic curtailed their PA, disrupted their routines, and revoked access to exercise programs, leaving them seeking new approaches to exercise. We found that our synchronous online exercise program was motivational and may reduce commonly reported environmental barriers, including removing concerns about transportation (eg, time spent traveling, driving, and parking), improving access through ease of participation from desired training locations, and removing poor weather condition concerns. Therefore, participants expressed a preference for synchronous online delivery of exercise programs, which may be a valuable option for motivating older adults to become physically active while reducing common environmental barriers.

Our findings suggested that participants believed in the health benefits of exercise. Participants described motivation to exercise due to their desire to be healthy, mobile, and participate in meaningful activities as they aged. Notably, the majority of participants in our study were previously active and had prior experience with exercise, which may influence their perceptions and experiences with exercise. Our findings are similar to findings from a qualitative study by Harrison et al [26] focused on understanding the barriers and motivators of 58 older adults (49/58, 84% females; 60‐85 years) residing in an urban community in Washington, DC, which suggested that the main benefits and reasons for exercising are prolonged life, more energy, and a stronger body. Other participants in our study reported their appearance as a motivator for exercise. While participants in our study discussed their motivations for exercising, they lacked confidence in their abilities to exercise safely, feared injury, and desired supervision. Participants described how traditional gyms lack adequate supervision and support for older adults learning how to exercise. Access to exercise programs specific to older adults is critical to help motivate individuals to exercise and maintain PA levels [5,6].

Our study was conducted during the COVID-19 pandemic, which has been shown to limit walking, biking, PA, and mobility while increasing sedentary behavior across all age groups [27]. As a result, participants discussed the interruption of the pandemic to their lives and its impact in our interviews. While some evidence suggests that older adults became more active in the pandemic [28], this was not the case for our participants, who reported experiencing changes to their PA levels and daily routines and found it difficult to motivate themselves to be physically active independently. Similarly, in a 2021 qualitative study by Petersen et al [29], 12 healthy Canadian adults (6/12, 50% females; 20‐70 years), participants described disruptions to their daily routines and changes in PA as a result of the pandemic. Our study builds upon these findings by providing insight into how the closure of gyms and canceled exercise programs impacted participants in our study, as many were active members before the pandemic. Limited access to exercise programs may lead to difficulty for older adults in motivating themselves to be physically active.

Social connectivity is a key motivator for older adults to maintain adherence to exercise programs [6,30]. Our findings suggest that synchronous online exercise programs are motivational for older adults as they can connect in real time with instructors and other participants, which is unique to synchronous programming compared with asynchronous programs as older adults identify the lack of real-time objective feedback [31]. However, some older adults in our study had previously noted that socializing may not be important when exercising, and they prefer to focus on exercising alone [17]. The synchronous program made our participants feel comfortable exercising when they could not attend in-person programs, and it was advantageous compared with in-person exercise as they did not need to share their space. Synchronous programs may be a useful exercise approach as many older adults are shifting towards more digital connections with family and friends beyond the pandemic, as seen in a sedentary behavior reduction intervention with older adults [32]. However, this may not be a generalizable finding, particularly for older adults in Canada with lower socioeconomic status and lower educational attainment, as there may be barriers to technological access and literacy [33,34]. Careful consideration is needed to develop accessible future interventions and programs when leveraging technology to deliver synchronous PA and exercise programs more broadly. Future work should consider using in-person recruitment to ensure greater diversity in studies [35].

In addition to technology barriers, other commonly cited barriers to participation in PA and exercise included environmental factors, access to resources, assistance in managing change, and social influences for older community-dwelling adults [5,6]. Our findings reflect the environmental barriers reported in the literature, including transportation, access to exercise facilities, and poor weather conditions. Participants in our study expressed concerns about driving and parking at fitness centers, but these transportation concerns were alleviated through our online exercise program, which enabled participants to focus on exercising. Participants described how the “Internet” was a good way to “deal” with transportation concerns, as many described the extra mental load of in-person programming. Previous research has highlighted the importance of transportation assistance in promoting the uptake of programs and maintenance of PA for community-dwelling older adults who are socially disadvantaged or experience disability [36-38]. Reducing transportation concerns may improve the accessibility of exercise programs and the ability for older adults to participate from their desired training location [38].

Barriers to exercise accessibility due to lack of time (family and work) and lack of facilities are other commonly reported environmental barriers [5,6]. In focus groups with recently retired older adults, they described feeling like they are relied upon by children, grandchildren, parents, and friends, making it difficult to prioritize structured PA [39]. For some, online programs provide convenience in terms of not needing transportation, allowing older adults to spend more time on other priorities [40]. Our participants commented on the ease of getting up 5 minutes before class and our program’s convenience. The frequency of our program, 3 times per week, was also feasible for older adults [17]. Our synchronous online exercise program improved participants’ access to exercise specific for older adults and allowed the flexibility of attending class from home while still remaining socially connected. It additionally created a psychologically safe place for participants compared with traditional gyms, which are dominated by younger adults and lack supervised training specifically for older adults. Creating convenient, safe, and synchronous programs is important for ongoing adherence [39].

Poor weather conditions (eg, winter, rain, extreme heat) in Canada are another environmental barrier that contributes to increased sedentary time [41]. Our findings suggest that ice, snow, rain, and extreme heat discourage exercise, particularly since most older adults must travel to a facility to participate in an exercise program. Participants suggested that online delivery may effectively reduce this environmental barrier as participants do not need to be concerned with “cleaning their cars off” or “walking on ice” to attend a training facility to exercise. In older Canadians, there is a decrease in PA levels during precipitation [42] and winter months [8]. Synchronous delivery of exercise may be a feasible approach to deliver programs broadly, particularly to populations who may not access in-person programs easily. Future work should explore hybrid exercise programming models, including in-person and synchronous delivery.

### Strengths and Limitations

Our study suggests that synchronous exercise programs are motivational for older adults as they can connect in real-time with instructors while eliminating common environmental barriers to in-person exercise programming. Interpretive design allowed us to address a complex experiential question while producing a practical outcome [19]. A potential limitation of this study is the limited diversity among our sample. Our study population was predominately Caucasian, with high education attainment, women between 65 and 80, and motivated to exercise. Thus, the findings do not represent the perceptions and experiences of structurally marginalized populations, those experiencing lower socioeconomic status, men, older than 80 years, and those with low levels of education attainment or motivation to support behavioral change. We did not include nonparticipants in the program (n=1) in the interviews. Including nonparticipants may have allowed us to elucidate further barriers and health inequities for those unable to complete the program. Although the study lead provided technological support and assistance through Zoom or a phone call, including turning on cameras, muting and unmuting, and connecting speakers, all participants had good technology literacy and digital access. Future studies should consider including individuals in different populations with varying degrees of technological literacy. Additionally, conducting the synchronous online exercise program outside of the COVID-19 context would provide valuable insight into the long-term feasibility of this program. Trustworthiness may not have been achieved as the transcripts and codes were not checked and confirmed by the participants in this study [25]; however, triangulation across analysts strengthened this aspect of rigor.

### Conclusion

The delivery of exercise programs using synchronous online classes may help older adults meet PA guidelines. Older adults report experiencing environmental barriers, including transportation, lack of access, and poor weather conditions when exercising. Our findings suggest that synchronous online exercise programs may serve as an approach to mitigate these environmental barriers and motivate older adults while keeping them socially connected. Considerations for designing exercise programs for older adults include delivery of the program by exercise and health professionals, synchronous programming to maintain social connectivity, and reducing environmental barriers such as transportation and weather concerns. Community programs may consider implementing synchronous online exercise programs as part of their recreational programming for older adults to increase engagement and reduce accessibility barriers. Future work should focus on leveraging synchronous exercise programs in community programs to engage older adults in PA and exercise and explore hybrid (synchronous and in-person) options.

## Supplementary material

10.2196/66473 Multimedia Appendix 1 Semistructured interview questions.

## Abbreviations

CON: control group
PA: physical activity
RCT: randomized controlled trial
