# Supplementary material for: Older Adults’ Perspectives on Participating in a Synchronous Online Exercise Program: Qualitative Study
Source: JMIR Aging. 2025 Apr 3;8:e66473. doi: 10.2196/66473 (PMC11984789; doi:10.2196/66473)
Supplement: Multimedia Appendix 1 [file aging-v8-e66473-s001.docx]

| **Appendix 1.** Semi-structured interview questions. |
| --- |
| **General questions**   1. Why were you interested in participating in this research? 2. Have you participated in a group-based physical activity program prior to participating in this study? 3. Can you tell me about what motivates you to participate in physical activity? 4. What kind of challenges or barriers do you experience when participating in physical activity? 5. Do you intend to continue to participate in physical activity? 6. Do you have any other comments to make about engaging in physical activity before we move on to talk about the exercise program? |
| **Perceptions of the Exercise Program**   1. Can you tell me a bit about the things that you liked about the exercise program? 2. Was there anything that you didn’t like about the program, or that you would change? 3. It can sometimes be a challenge to participate in physical activity. What were some of the things that helped you to stay motivated? [Prompt for: online classes, live classes, instructors] 4. Was there anything that made it difficult for you to participate in the exercises or the program? 5. Do you have any suggestions for improving the program or improving the support that is offered that help participants to engage in physical activity? 6. If we were to host another series of sessions, what type of classes or exercises would you like to see? 7. Do you have any other comments you’d like to make about the exercise program before we move on? |
| **Perceptions of the Research Study**   1. In general, what did you think about having to complete all of those surveys? Did you find them easy to complete or was it a burden? 2. Do you have any feedback for us about how the surveys were set up, in terms of how clear or understandable they were, or how long they were? 3. Do you have any suggestions for how we might improve this? 4. Do you have any other comments to make about the surveys or the research study in general? |
